# The effect of anti-frustration ability on academic frustration among Chinese undergraduates: A moderated mediating model

**DOI:** 10.3389/fpsyg.2023.1033190

**Published:** 2023-02-09

**Authors:** Minru Wu, Hua Huang, Yuanshu Fu, Xudong Zhang

**Affiliations:** ^1^School of Psychology, South China Normal University, Guangzhou, Guangdong, China; ^2^School of Education, Zhaoqing University, Zhaoqing, Guangdong, China

**Keywords:** anti-frustration ability, academic frustration, core competence, coping style, Chinese undergraduate

## Abstract

**Background:**

With the ongoing push to improve the quality of talent in all professions, academic pressure on undergraduates is gradually increasing, leading to students feeling increasingly frustrated by academic stressors. As it becomes more widespread, the resulting issue of academic frustration is attracting public attention.

**Aim:**

The current study explored the relationship between undergraduate anti-frustration ability (AFA) on their academic frustration (AF), focusing specifically on the roles played by core competence (CC) and coping style (CS) in this relationship.

**Methods:**

Our sample comprised 1,500 undergraduate students from universities in China. Data collection made use of the Ability to Anti-Frustration Ability Questionnaire, the Academic Frustration Questionnaire, the Core Competence Questionnaire, and the Simple Coping Style Questionnaire.

**Results:**

The results showed that: (1) AFA negatively predicted the AF of undergraduates, with CC mediating this relationship and (2) CS had a moderating effect on the relationship between CC and AF. We concluded that students who employ positive CS may be more successful in alleviating their AF to a larger extent, with the mediation of CC.

**Interpretation:**

The results revealed the mechanism of AFA on AF, which will help schools consider and guide students’ skills and abilities, both academically and personally.

## Introduction

Academic frustration (AF) is defined as a negative psychological emotion experienced by students when they are unable to deal with frustration in their learning process (Ballmann et al., 2022). AF can negatively impact students’ interest and enthusiasm in their academic activities, causing them to feel isolated due to their perceived failures, thereby further lowering their personal achievement motivation (Neff et al., 2005). Although AF is a negative emotional experience, it may sometimes lead to positive outcomes. For example, negative emotional experiences can have an amplification function whereby individuals become more motivated to address their own problems with purpose, which is conducive to targeted problem solving (Mather et al., 2015; Xie and Zhang, 2016). Nonetheless, confusion and frustration in academic activities may enhance students’ learning effect in the short term, however the feeling of frustration nevertheless is detrimental on the long-term learning effect (Peddycord-Liu et al., 2013). In order words, while AF may sometimes be helpful, it does appear to harm study outcomes in the long run.

Thus far, little research has directly investigated specifically students’ AF. Instead, studies have focused primarily on some of the leading factors that might cause AF, such as academic failure or level of frustration tolerance. For example, Perry et al. (2005) found that students’ academic failure is intimately related to their perceived academic control. Those with high academic control pay little attention to academic failure, which leads to little AF. Harrington (2005) found that students with different levels of frustration tolerance might adopt different strategies to deal with stress during the learning process to relieve frustration. Several studies have pointed out that anti-frustration ability (AFA) is a crucial factor causing negative academic emotions, e.g., academic frustration (see Ou et al., 2013; Yang and He, 2018; Yang et al., 2021). The concept of AFA may be derived from frustration tolerance (Yang and He, 2018). While frustration tolerance described one’s ability to *bear* or *withstand* the pressure when encountering setbacks, AFA emphasizes the ability to not only *endure* frustration but to *grow* with frustration, and *take action* against frustration (Yang and He, 2018). Wang et al. (2006) have argued that people with high resilience, that is, those who possess high AFA, tend to experience more positive emotion. When in moments of frustration, individuals with high AFA are able to employ different strategies such as self-deprecation or humor to produce more positive feelings, alleviate stress, and adapt to the environment (Block and Kremen, 1996; Tugade and Fredrickson, 2004). Dialectical behavior therapy (DBT) contends that the tolerance of pain, in this case frustration tolerance, plays a central role in one’s ability to adapt to undesirable behaviors and alleviate frustration (Simons and Gaher, 2005). Frustration tolerance is the reflection of students’ view and attitude toward AF (Li et al., 2019). However, each person adapts to frustration differently. People with strong frustration tolerance can withstand stressors and manage to find their way through periods of learning difficulties (Li et al., 2019). As studies indicate that AFA might play an important role in college students’ ability to deal with their AF, we thus proposed our first hypothesis: AFA directly influences students’ AF (H1).

Theoretically, AFA can trigger positive emotion—which is initially constructive—and thus broaden an individual’s attention and cognition (Fredrickson, 2003). This facilitates one’s ability to enrich their persistent resources. In this process, core competence (CC, or key competence) is regarded as a kind of crucial persistent resource that might work on AFA’s influence upon AF. CC is the essential character and crucial competencies that enable students to adapt to the changing world (Wiek et al., 2011). CC facilitates the development of one’s creativity, self-directedness, and self-motivation (Xin et al., 2016). It also helps students to deal with various challenges, including those they face in their academic activities. Scholars tend to regard CC as an important part of the fundamental dynamics that facilitate a student’s ability to adapt to the future world (Lin, 2016). According to Huang et al. (2016), CC is multi-layered, involving culture-related literacy (e.g., language, mathematics, science), self-related literacy (e.g., learning skills, mental health, self-management), and society-related literacy (e.g., social responsibility, value, beliefs). Guerra and Bradshaw (2008) have pointed out that youth should develop five core competencies including (1) a positive sense of self, (2) self-control, (3) decision-making skills, (4) a moral system of belief, and (5) prosocial connectedness. The cultivation of undergraduates’ core qualities is a key requirement of the connotative development of higher education, as well as an important way to carry out the fundamental task of establishing morality and cultivating talents and developing socialist builders and successors with all-round development of morality, intelligence, physique, and esthetics (Lin, 2016). With the ongoing evolution of educational resources, both conceptually and tangibly, undergraduates are being pushed to perpetually improve their individual comprehensive abilities, to achieve an increased comprehensive development in their studies and personal life. A study conducted in Chinese high schools found that students’ AFA was significantly positively related to CC (Li et al., 2020), while Xing et al. (2018) showed that AFA can impact students’ CC development, and CC, in turn, helps students deal with difficulties in their academic activities. Based on these findings, we proposed our second hypothesis: CC plays a mediating role in students’ AFA’s influence on their AF (H2).

Coping style (CS) in the current study is defined as one’s cognitive and behavioral efforts utilized to help one adapt to their environment (Lazarus, 1993). Depending on its impact on one’s mind and body, CS can be both positive and negative. Positive CS are problem-centered, that is, the individual tries to solve practical problems, while negative CS are emotion-centered, that is, individuals reduce their negative emotions by using coping strategies such as avoidance and denial (Di et al., 2015). Regarding the theory of CS, contextualism holds that one’s CS can vary across different situations (Furtado et al., 2016). For example, in the context of academic difficulties, the CS that one adopts will depend on their evaluation of controllable events and the resources they can access to manage stress (Furtado et al., 2016; Su et al., 2021). Positive or adaptive strategies decrease the amount of stress one perceives and experiences, while negative or maladaptive strategies diminish symptoms of stress but without addressing the real problem or disorder. When employing a positive CS, individuals are able to mobilize more psychological resources, including CC (Matud, 2004). Li et al. (2020) found that CC was closely related to positive CS: individual with high CC tended to employ a positive CS during moments of difficulty. Okechukwu et al. (2022) showed that those who employ a positive CS are better able to deal with their AF. Moreover, a positive CS enables people to experience happiness and pride in their learning, both of which can increase their academic motivation and interest while decreasing their AF (Fitzgibbon and Murphy, 2022). Based on these findings, we proposed our third hypothesis: CS can be viewed as a moderator in CC’s relationship with AF (H3).

**Figure 1:**
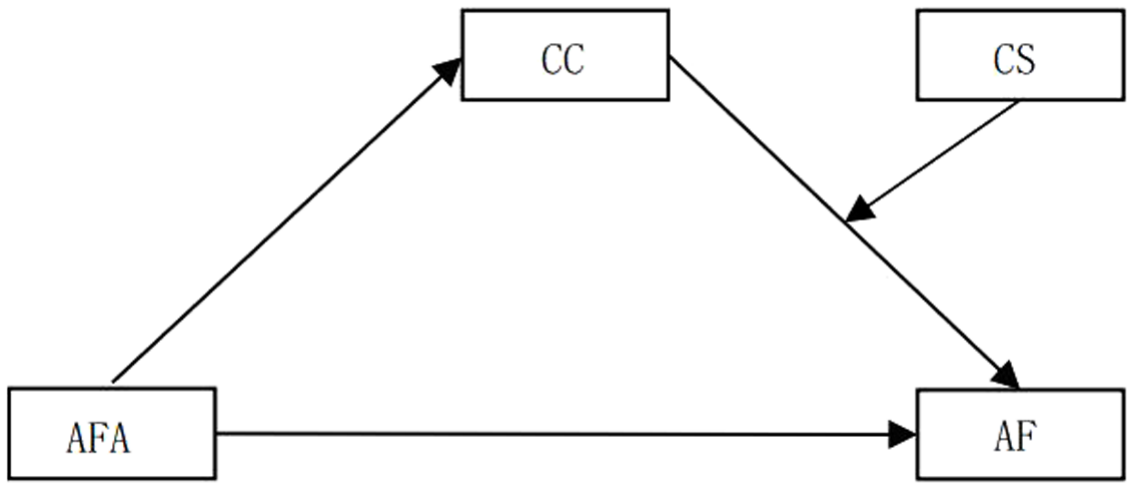
A moderated mediation model for AFA and AF.

To sum up, the present study constructed a moderated mediation model (Figure 1) in which AFA impacts students’ AF through the mediating role of CC, while CS also moderates CC’s effect upon AF. This model provides a heuristic framework to understand how and when students’ AF is affected by their AFA.

## Methods

### Participants

Our study sample was made up of undergraduate students studying in China. We sent out 1,800 online questionnaires *via* Sojump^1^ to undergraduate students at 32 different universities in China, covering 18 provinces. The sampling was incidental, due to accessibility. A total of 1,500 undergraduates ultimately took part in the research. All participants gave informed consent before filling out the questionnaire. Of the full sample, 55.3% were male (*n* = 829) and 44.7% were female (*n* = 671). The age of the participants ranged from 16 to 25 years. More information on the composition of the study sample is shown in Table 1.

**Table 1:** Sample characteristics (*n* = 1,500).

	*n*	%
*Gender*		
Male	829	55.3
Female	671	44.7
*Grade*		
Freshman	584	38.9
Sophomore	623	41.5
Junior	241	16.1
Senior	52	3.5
Hometown area		
Urban	474	31.6
Rural	1,026	68.4
Academic performance		
In the top 1/3 of the class	512	34.1
In the middle 1/3 of the class	776	51.7
In the bottom 1/3 of the class	212	14.1

### Measures

#### Anti-frustration ability questionnaire

Developed by Zhang (2013), this questionnaire using a total of 48 items to measure 10 factors. These factors are frustration tolerance, frustration resilience, frustration experience, career programming ability, confidence, interpersonal interaction ability, frustration understanding ability, psychological preparation, attribution ability, and attribution ability. Respondents rate each item using a five-point scale, ranging from 1 (completely disagree) to 5 (completely agree). A high total score indicates high anti-frustration ability. Cronbach’s α for the total questionnaire in the present study was 0.95.

#### Academic frustration questionnaire

This questionnaire was developed by Zhang (2020), and uses 41 items to measure six factors. These factors include learning motivation frustration, learning environment frustration, test frustration, learning stress frustration, learning adaptation frustration, and learning confidence frustration. Respondents rate each item using a five-point scale, ranging from 1 (completely disagree) to 5 (completely agree). A high total score indicates a high level of academic frustration. Cronbach’s α for the total questionnaire in the present study was 0.92.

#### Core competencies questionnaire

This questionnaire is based on Lin’s (2017) construct of Chinese students’ core development literacy, and further revised by Zhang (2020). The questionnaire uses 43 items to measure nine factors: literacy achievement, esthetic sentiment, rational thinking, enjoyment of learning, self-reflection, self-management, national identity, national understanding, and problem-solving. Respondents rate each item using a five-point scale, ranging from 1 (completely disagree) to 5 (completely agree). A high total score indicates a high core competence. Cronbach’s α for the total questionnaire in the present study was 0.96.

#### Simple coping style questionnaire

The simple coping style questionnaire (SCSQ) was developed by Xie (1998) and comprises 20 items. Twelve items measure positive coping, and eight items measure negative coping. Respondents rate each item using a four-point scale, ranging from 1 (completely disagree) to 4 (completely agree). As we were considering positive and negative coping styles separately, we calculated the Cronbach’s *α* on each of these two dimensions. The Cronbach’s *α* for the two dimensions of the questionnaire in the current study was 0.76 and 0.78, respectively.

### Statistical processing

The current study used SPSS 22.0 and the PROCESS macro provided by Hayes (2012) for the data analysis.

### Testing for common method biases

The present study adopted Harman’s single-factor test to examine common method biases. Exploratory factor analysis showed that 31 factors had an eigenvalue higher than 1. The first factor explained 16.01% of the total variation, far less than the cutoff value of 40% (Podsakoff et al., 2003). Therefore, no apparent common method biases were evident in the present study.

## Results

### Description and correlational analysis

As shown in Table 2, academic frustration was significantly negatively related to anti-frustration ability and positive coping style (*p* < 0.001), and significantly positively related to core competence and negative coping style. Core competence was positively related to positive coping style and negatively related to negative coping style.

**Table 2:** Descriptive statistics of variables and their correlations (*n* = 1,500).

	*M* ± *SD*	1	2	3	4	5
(1) Anti-frustration ability	3.52 ± 0.56	–				
(2) Academic frustration	2.77 ± 0.54	−0.38^***^	–			
(3) Core competence	3.25 ± 0.61	0.28^***^	−0.34^***^	–		
(4) Positive coping style	1.87 ± 0.43	0.36^***^	−0.19^***^	0.35^***^	–	
(5) Negative coping style	1.34 ± 0.56	−0.20^***^	0.25^***^	−0.13^***^	0.17^***^	–

^***^p < 0.001.

### Testing for moderated mediated model

**Figure 2:**
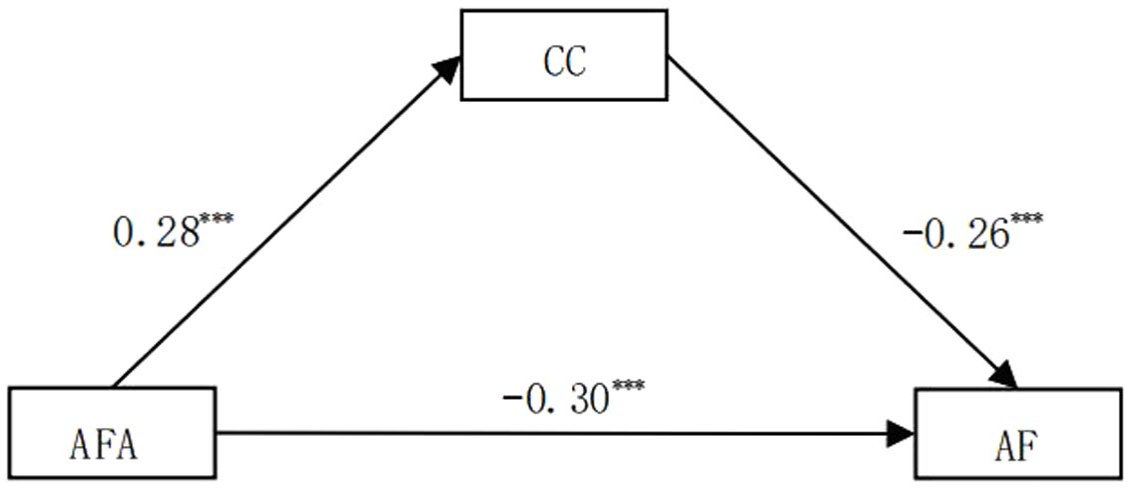
Core competence (CC) as the mediator between AFA and AF.

Using the standardized variables, we used the SPSS PROCESS macro by Hayes (2012) to analyze core competence as a mediator between anti-frustration ability and academic frustration. The linear regressions are shown in Table 3, and the mediating model is shown in Figure 2.

**Table 3:** Predictors for academic frustration using core competence as mediator.

Variables	Academic frustration		Core competence		Academic frustration	
	*β*	*t*	*β*	*t*	*β*	*t*
Anti-frustration ability	−0.38	−15.73^***^	0.28	11.37^***^	−0.30	−12.63^***^
Core Competence					−0.26	−10.77^***^
*R^2^*	0.14		0.08		0.20	
*F*	247.47^***^		129.27^***^		191.17^***^	

^***^p < 0.001.

Table 3 shows that AFA can significantly positively predict core competence (*β* = 0.28, *t* = 11.37, *p* < 0.001) and negatively predict academic frustration (*β* = −0.30, *t* = −12.63, *p* < 0.001). Furthermore, core competence significantly negatively predicts academic frustration (*β* = −0.26, *t* = −10.77, *p* < 0.001).

A non-parametric percentile bootstrap was conducted to examine the mediating effects, as shown in Table 4. Bias-corrected 95% confidence intervals on 5,000 bootstrap samples were estimated for all tests of the total effect, direct effect, and indirect effect. All of the 95% CIs did not include zero, which suggests that AFA could affect AF both directly and indirectly through the mediating role of CC.

**Table 4:** Core competence as a mediator between AFA and AF (5,000 bootstrap samples).

Effect type	Effect value	BootSE	95%CI
Total effect	−0.38	0.03	[−0.43, −0.33]
Direct effect	−0.30	0.03	[−0.35, −0.26]
Indirect effect	−0.08	0.01	[−0.09, −0.05]

Model 4 of the PROCESS macro (Hayes, 2012) was used to examine CS as the moderator. CS was estimated by calculating the difference of the standardized positive and negative coping scores. A positive difference value suggested that one was moving toward adopting a positive coping style, while a negative result suggested one was moving to adopt a negative coping style (Dai, 2010). The results indicated that the combined predicting effect of CC and CS on AF was statistically significant (Table 5).

**Table 5:** Testing for moderated mediation model.

Variables	CC		AF	
	*β*	*t*	*β*	*t*
AFA	0.28	11.37^***^	−0.24	−9.43^***^
CC			−0.22	−8.64^***^
CS			−0.12	−5.74^***^
AFA × CS			−0.04	−2.03^*^
*R^2^*	0.08		0.22	
*F*	129.27^***^		107.09^***^	

^*^p < 0.05,

^***^p < 0.001.

**Figure 3:**
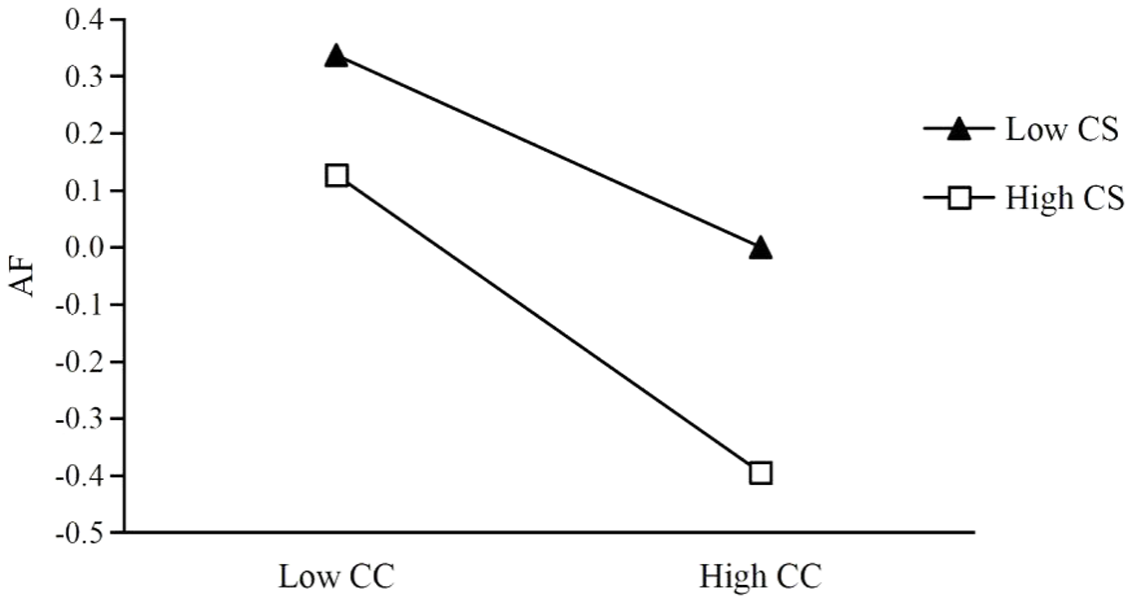
Coping style (CS) as the moderator among CC and AF.

To further understand the interacting effects of CC and CS on AF, we followed Fang et al.’s (2015) procedure with regard to conducting a simple slope analysis on significant interaction. First, we labeled high CS and low CS groups according to whether the participant scored higher or lower than the standardized mean. Then, the simple slopes of their CC predicting AF of the two groups were calculated separately. The results are shown in Figure 3.

In both groups, CC was shown to negatively predict AF. However, the predicting effect size of the high CS group (*β* = −0.26, *t* = −8.06, *p* < 0.001) was much larger than that of the low CS group (*β* = −0.17, *t* = −4.81, *p* < 0.001). This indicated that adopting a positive coping style could enhance the effect size of CC in predicting AF, which suggested a mediating effect of CC between the undergraduates’ AFA and AF (Table 6).

**Table 6:** Different mediating effects of different CS.

	CS	Effect value	BootSE	95%CI
The mediating effect of CC	−1.29(*M* – 1*SD*)	−0.05	0.01	[−0.07, −0.03]
	0.00(*M*)	−0.06	0.01	[−0.08, −0.04]
	+1.29(*M* + 1*SD*)	−0.07	0.01	[−0.10, −0.05]

## Discussion

### The mediating effect of core competence

The present study first showed that AFA could facilitate undergraduates’ ability to deal with their AF, replicating results of previous studies (e.g., Li et al., 2019; Yang et al., 2021), thus verifying H1. AFA is a psychological resource that functions as a protective factor, e.g., induces more positive emotions (Wang et al., 2006). According to the broaden-and-build theory (Fredrickson, 2013), positive emotion allows individuals to broaden their attention, cognition, and behavior, enabling them to more keenly observe the existing world and more effectively observe and analyze relevant information during moments of difficulty (Contractor et al., 2021). Positive emotions help individuals increase their achievement goals related knowledge and experience, thus inspiring newer models of thought, developing new problem-solving models, and allowing them to adopt a positive CS in order to deal with frustrations (Wolkenstein et al., 2022). Positive emotions also facilitate the correction, adjustment, and dispelling of the prolonged impact of negative emotions. Therefore, students with higher levels of AFA tend to have fewer negative emotions, e.g., AF.

Furthermore, our findings showed that AFA could not only directly affect AF, but that it also had an impact through the effect of CC. In other words, AFA positively predicted undergraduates’ CC, and CC positively predicted their AF, thus verifying H2. The results in the present study indicate that CC partly mediates the relationship between undergraduates’ AFA and their AF. CC is the essential and stable ability that enables one to adapt to the changing world around them, which provides them with long-term supportive strength (Wiek et al., 2011). The mediation model of CC could also be explained by the broaden-and build theory: positive emotions induced by AFA allows students to build and strengthen their sustainable resources, e.g., CC, which would become an integral part of future actions and relieving their levels of stress or frustration (Fredrickson, 2013; Peng et al., 2014). In the midst of difficulties, one who tended to focus on the events of frustration would narrow their attention and cognition, limit their behavior, and lead to admitting their failure and feelings of hopelessness, thus become deeply trapped in frustration. To alleviate the prolonged effect of frustration, then, individuals can engage AFA, such as seeking out broader resources (e.g., CC) in an effort to support themselves. Positive emotion, broadmindedness, and the development of one’s resources all work together interactively. For example, positive emotion can broaden one’s attention and cognition, which thereby benefits them by allowing them to better deal with challenges and strengthen their resources which, in turn, enables them to further develop their positive emotions (Gao and Tong, 2010).

Specifically, CC plays an important role in alleviating undergraduates’ AF. When undergraduates transition to university, they gain unprecedented freedom and independence, but this means that they must learn to make their own decisions and career plans and learn to manage themselves effectively. Students with high levels of CC tend to form clear learning goals before beginning learning activities, develop a detailed study plan in preparation of the learning, and integrate self-monitoring and feedback to adjust and adapt their learning process and methods (Guo and Ji, 2019; Usán et al., 2022). Students with high levels of CC are able to make positive choices while having strong planning and execution skills, allowing them to move forward toward established goals thanks to their strict self-discipline (Wang and Zhao, 2018). Meanwhile, students with low levels of CC often manifest as poor self-control, inability to concentrate on learning, and low learning efficiency, all of which lead to unsatisfactory study results and a greater feeling of learning pressure. In the face of exam failure and academic difficulties, it quickly becomes difficult to adjust one’s emotions and mentality in a timely fashion, leading these undergraduates to become prone to a high level of AF. Therefore, undergraduates with high CC will experience less AF.

To relieve AF, Veronen and Kilpatrick (1982) stated that the stress inoculation training (SIT) could improve AFA and effectively alleviate one’s level of AF. According to Veronen and Kilpatrick (1982), the SIT operates from artificially creating a frustration situation and continuously enables students to overcome frustration under the guidance of authority (such as teachers), creating an invisible antibody to frustration psychologically. Meanwhile, AF could also be relieved with the improvement of CC, which could be achieved by setting clear learning goals, making full use of resources from the outside world, etc.

### Coping style as moderator

Our results indicate that CS plays a moderating role in the latter half of the mediating model: AFA → CC → AF. In other words, CS moderated the relationship between CC and AF. Specifically, students with a positive CS will generally experience a lower level of AF in comparison to those with a negative CS. Nevertheless, those with low CC will experience a higher level of AF if they concurrently employ a negative CS. In contrast, those with high CC will experience the lowest level of AF if they simultaneously employ a positive CS. A possible reason for this might be that a negative CS tends to result in the individual losing confidence and feeling powerlessness, which limits the function of their CC and makes them vulnerable to the trap of AF.

Students with negative CS tend to deal with their problems by lowering their expectations and self-handicapping. This further inhibits their CC, and their negative coping abilities and inadequate resources to handle stressors further exacerbate their frustration and weaken their resilience. However, positive CS helps individuals experience more positive emotions when confronted with negative events, allowing them to evaluate events in a more positive way, buffering potential negative impacts (Yin et al., 2010). Those with a positive CS tend to adopt more effective problem-solving tactics and seek help from others, leading to better-functioning CC as they deal with frustration and stress, thereby assisting them in reducing potential negative emotions triggered by AF. Positive CS has a psychological construction effect that can trigger more positive emotion and help one recover psychological resources inhibited by frustration (Noorbakhsh et al., 2010). In other words, the essence of positive CS is to utilize the possible resource to buff the negative effect of frustration and, in this process, CC functions to amplify and provide the supportive energy. Even individuals with low CC may benefit from adopting a positive CS, as it can to some extent further boost their AFA.

There is a never-ending need for new talent and CC that integrates values, attitudes, emotions, skills, and knowledge is essential for students’ development, helping them adapt to changing social settings and achieve success. The cognitive interaction theory of stress indicates that one’s choice of CS is influenced by cognitive evaluation. When facing a stressful situation, one should consider whether they can effectively overcome the stressors based on their own personal reserves and resources, while also taking into account their capabilities and style of problem-solving as well. When one fears their personal resources may not be strong enough to cope with external threatening events, they have a tendency to fear the potential difficulties ahead and, in response, adopt more negative coping methods such as avoidance or evasion responsibility (Zhu et al., 2022). According to the theory, undergraduates with stronger CC are more likely to have stronger all-round development, and be more likely to adopt positive coping methods in the face of difficulties and stress. Those adopting positive coping strategies will tend to deal with the problem at hand rather than try to escape it or fall into emotional distress. If undergraduates learn to adopt task-oriented coping methods, they will likely suffer less frustration in their studies, and therefore achieve better academic performance (Khorasani and Ghanizadeh, 2017).

## Limitations and future research

Despite the contributions of our findings to the current understandings of the mechanisms at play between AFA and AF, there are nonetheless some limitations to this study. First, the different grade demographics in the sample was not balanced. Most of the sample was made up of freshmen and sophomores, which can have been a result of the random distribution of questionnaires. Also, the sampling of academic performance was not balanced. Most of the sample were in the middle third of the class in terms of academic achievement. This could have been due to random sampling, or to the “*zhongyong*” (Doctrine of the Mean, which prefers harmony and avoids extremities, see Yang et al., 2016; Fan, 2021) nature of the Chinese. Future studies should adopt a more rigorous design, and consideration of how to distribute the questionnaires effectively should be determined before beginning the study, so as to improve the data quality. Second, the four scales contained a relatively large number of questions, and respondents may have grown tired before completing all questionnaires, which may have led to errors between the measured data and reality. Future research should endeavor to develop shorter but nonetheless effective questionnaire forms. Third, data analysis techniques in this study were limited to several common traditional analysis methods. In the future, similar studies should explore the data further and deeper. Last but not least, the concepts of AF, AFA, and CC are all relatively new at this point, so there is a lack of appropriate literature explaining the relationship between these, as well as the mechanisms at play between variables. However, we considered this to be an important innovation of the current study, to put forward these concepts. Furthermore, we welcome future studies in the same vein for further validation of our results.

## Conclusion

To the undergraduates, their AFA might lead to alleviating AF and this process is partly mediated by CC. In comparison with negative CS, positive CS employed by undergraduates might help alleviate their AF to a larger extent with the mediation of CC.

## Data availability statement

The raw data supporting the conclusions of this article will be made available by the authors, without undue reservation.

## Ethics statement

The studies involving human participants were reviewed and approved by XZ, Zhaoqing University. The patients/participants provided their written informed consent to participate in this study.

## Author contributions

MW: investigation, data analysis, and original draft. HH: conceptualization, data collection, and translation. YF: conceptualization, data analysis, and supervision. XZ: data collection, and review and editing. All authors contributed to the article and approved the submitted version.

## Funding

This project was funded by Program of the National Social Science Foundation of China (grant no. BBA180078).

## Conflict of interest

The authors declare that the research was conducted in the absence of any commercial or financial relationships that could be construed as a potential conflict of interest.

## Publisher’s note

All claims expressed in this article are solely those of the authors and do not necessarily represent those of their affiliated organizations, or those of the publisher, the editors and the reviewers. Any product that may be evaluated in this article, or claim that may be made by its manufacturer, is not guaranteed or endorsed by the publisher.

## Footnotes

^1^ http://www.wjx.cn
